# Triumphs of Mechanical Surgery

**Published:** 1864-05

**Authors:** 


Triumphs of Mechanical Surgery.—Mechanical dentistry is but another term for mechanical surgery. It remedies congenital or acquired defects of the oral cavity by mechanical appliances. To the scientific conservative surgeon no branch of his art is more important than that which aims to accomplish, by nicely adjusted apparatus, what was formerly attempted by an operation, or to supplement the deficiencies of that art. An interesting instance of the value of scientific mechanical dentistry has been brought to our attention, and its triumphs over the best devised operation is most marked. The restoration of congenital cleft palate has always proved a most unsuccessful surgical operation. Even if the fissure was successfully closed the defect of speech was not improved materially. Dr. Kingsley, a dentist of this city, has, for several years made cleft palate a subject of special study, and his labors have been rewarded with success. Dr. Stearns, of the U. S. Army, who, in his own case adapted an apparatus which completely relieved the defect, was the first in this field. But his apparatus was never successfully applied in other cases. Dr. Kingsley has effectually completed the work undertaken by Dr. Stearns. His method is to take a mould of the defective parts, and to this adapt the instrument. The material is vulcanite. We have seen several persons wearing this apparatus, and the improvement in the speech was most satisfactory. The invention of Dr. K. must be regarded as a great triumph of mechanical surgery.—*N. Y. Med. Times.*

